# Effect of alpha-lipoic acid supplementation on polycystic ovary syndrome clinical outcome in infertile females treated with letrozole: a randomized controlled trial

**DOI:** 10.1007/s44446-026-00084-0

**Published:** 2026-08-19

**Authors:** Moaz Ahmed Sallam, Haitham Hamza, Sara Mahmoud Shaheen, Marwa Adel Ahmed

**Affiliations:** 1 Pharmacy Practice Department, Faculty of Pharmacy, Sinai University, Ismailia, Egypt; 2 Gynecology and Obstetrics Department, Faculty of Medicine, Menoufia University, Menoufia, Egypt; 3 Clinical Pharmacy Department, Faculty of Pharmacy, Ain Shams University, Cairo, Egypt

**Keywords:** Letrozole, Alpha lipoic acid, Oxidative stress, Ovulation induction, IR, Polycystic ovary syndrome

## Abstract

Non-responsive cycles during ovulation induction with letrozole (LZ) often necessitate the use of human menopausal gonadotropin (HMG), which may be associated with undesirable adverse effects. Therefore, strategies to improve the therapeutic outcomes of LZ are needed. This trial aimed to evaluate the impact of combining alpha-lipoic acid (ALA) with LZ on ovulation induction in women with polycystic ovary syndrome (PCOS). This randomized controlled trial included 151 infertile women with PCOS who were randomly allocated to either the LZ group (*n* = 76) or the ALA group (*n* = 75). The LZ group received LZ (2.5–7.5 mg) with lifestyle modification for up to three treatment cycles or until pregnancy occurred. The ALA group received the same regimen in addition to ALA (1800 mg/day), which was discontinued on the day of human chorionic gonadotropin (HCG) injection in ovulatory patients. Ovulation and pregnancy rates were assessed, along with hormonal parameters. Ovulation per cycle was significantly higher in the ALA group [153/166 (92.2%)] compared with the LZ group [146/203 (71.9%)] (*P* = 0.035). Moreover, pregnancy per cycle was significantly higher in the ALA group than in the LZ group (*P* = 0.033). Although serum estradiol, mid-luteal progesterone, and endometrial thickness showed numerically higher values in the ALA group, the differences between the groups were not statistically significant after Bonferroni adjustment. The addition of ALA to LZ improved ovulation and pregnancy outcomes and was associated with favorable metabolic and hormonal effects in women with PCOS.

## Introduction

Polycystic ovary syndrome (PCOS) is the most common endocrine disorder affecting the ovaries and represents a major cause of infertility among women of reproductive age, with a global prevalence estimated at 9.2% (Salari et al. 2024). Infertility in women with PCOS is primarily attributed to ovarian dysfunction and irregular ovulation, which is often aggravated by insulin resistance (IR). Hyperinsulinemia associated with IR stimulates ovarian theca cells to produce excess androgens, thereby contributing to the complex pathophysiological cycle characteristic of PCOS (Purwar and Nagpure 2022; Zhu et al. 2024). In addition, several studies have reported elevated circulating oxidative stress markers in women with PCOS compared with healthy individuals, suggesting that oxidative stress may play a significant role in the development and progression of the disorder (Murri et al. 2013).

Letrozole (LZ), an aromatase inhibitor, suppresses estrogen synthesis by inhibiting the aromatase enzyme. Administration of LZ during the follicular phase reduces the negative feedback of estrogen on the hypothalamus and pituitary gland, thereby increasing gonadotropin secretion and stimulating follicular development (Guang et al. 2018). In recent years, LZ has been recognized as a first-line pharmacological treatment for ovulation induction in women with PCOS, comparable to clomiphene citrate (CC) (Kar 2012; Wang et al. 2019). Unlike CC, LZ does not exert anti-estrogenic effects on the endometrium, allowing adequate endometrial thickness to be maintained during treatment. Moreover, LZ has a relatively short half-life of approximately 45 h and is rapidly eliminated from the body, minimizing the risk of drug accumulation (Amer et al. 2017). Currently, LZ is the most commonly prescribed oral medication for ovulation induction in PCOS patients (Franik et al. 2022; Pundir et al. 2021; Wang et al. 2019). However, when used as monotherapy, a proportion of treatment cycles remain non-responsive (Amer et al. 2017; Mejia et al. 2019; Sharma et al. 2023; Shi et al. 2022). In such cases, human menopausal gonadotropin (HMG) is often administered, although its use may increase the risk of ovarian hyperstimulation syndrome (OHSS) and other adverse effects (Chen et al. 2024; Dai et al. 2023; Weiss et al. 2019; Xia et al. 2023). Therefore, strategies aimed at improving the therapeutic outcomes of LZ are warranted.

One potential strategy is the addition of alpha-lipoic acid (ALA), also known as thioctic acid or 1,2-dithiolane-3-pentanoic acid, a naturally occurring compound found in mitochondria. ALA functions as an enzymatic cofactor in the Krebs cycle and contributes to cellular energy metabolism. It also plays an important antioxidant role by regenerating other antioxidants, such as vitamins C and E, from their oxidized forms (Evans et al. 2002). In addition, ALA exhibits insulin-like activity and has been shown to improve glucose metabolism and reduce insulin resistance (Rochette et al. 2015; Evans and Goldfine 2000). Furthermore, ALA possesses anti-inflammatory properties through inhibition of nuclear factor-kB (NF-κB) translocation to the nucleus, which subsequently reduces the production of pro-inflammatory cytokines (Ying et al. 2011; Akbari et al. 2018; Guo et al. 2016; Li et al. 2015).

Several clinical studies have investigated the potential benefits of ALA in patients with PCOS. Masharani et al. administered controlled-release ALA (600 mg) for 16 weeks to six lean, non-diabetic women with PCOS and reported a 13.5% improvement in insulin sensitivity (Masharani et al. 2010). Similarly, Genazzani et al. demonstrated that ALA supplementation at a daily dose of 400 mg improved metabolic disturbances across different PCOS phenotypes, with particularly notable improvements in patients with a family history of diabetes (Genazzani et al. 2018). Moreover, Fruzzetti et al. reported that co-administration of ALA with inositol improved both metabolic and hormonal parameters in women with PCOS (Fruzzetti et al. 2020).

In general, ALA is well tolerated and associated with minimal side effects, such as mild nausea or itching (Koh et al. 2011). A systematic review and meta-analysis further demonstrated that ALA supplementation was not associated with any significant adverse events during treatment (all p > 0.05). Importantly, its safety profile remained favorable across various patient subgroups, including individuals with smoking habits, cardiovascular diseases, diabetes, pregnancy, neurological disorders, rheumatic diseases, severe renal conditions, and pediatric or adolescent populations (Fogacci et al. 2020).

Therefore, the present trial aimed to evaluate the safety and efficacy of combining ALA with LZ for ovulation induction in infertile women with PCOS. Specifically, the study assessed reproductive, hormonal, and metabolic outcomes, including cumulative ovulation rate, pregnancy rate, serum estradiol (E2), mid-luteal progesterone (mid-P), endometrial thickness, fasting blood glucose (FBG), body mass index (BMI), homeostasis model assessment of insulin resistance (HOMA-IR), and fasting insulin (FI). In addition, the safety and tolerability of ALA supplementation were evaluated among the study participants.

## Patient and methods

### Ethical approval

The study protocol was conducted in accordance with the principles of the Declaration of Helsinki and was approved by the Faculty of Pharmacy Ethics Committee, Ain Shams University (ACUC-FP-ASU). It is important to note that both the Faculty of Pharmacy Research Ethics Committee and Menoufia University Hospitals operate under the same institutional governance framework. The Faculty of Pharmacy Research Ethics Committee is an officially recognized institutional ethics committee authorized to review and approve clinical research conducted within affiliated hospitals.

The trial was registered at ClinicalTrials.gov under the identifier NCT06418347. All participants and their caregivers were fully informed about the study protocol, and written informed consent was obtained from each participant prior to enrollment. Participants were informed of their right to withdraw from the study at any time without any obligation.

### Study design and participants

This study was designed as a prospective, randomized, open-label controlled trial conducted at the Obstetrics and Gynecology Department of Menoufia University Hospitals between April 2024 and August 2025. Outpatients presenting with infertility for at least one year due to anovulation associated with polycystic ovary syndrome (PCOS) were screened for eligibility.

The inclusion criteria were as follows:

(i) diagnosis of PCOS according to the modified 2003 Rotterdam consensus criteria (ESHRE and Group 2004), defined as oligo- or anovulation secondary to PCOS along with at least one of the following: clinical or biochemical hyperandrogenism, or polycystic ovarian morphology (POM) detected by ultrasonography;
(ii) female age between 18 and 35 years;
(iii) male partners with adequate semen parameters;
(iv) presence of at least one patent fallopian tube and a normal uterine cavity;
(v) willingness of the patient to conceive.

Patients were excluded if they had medical conditions known to affect fertility, including thyroid disorders, congenital adrenal tumors, hyperprolactinemia, Cushing’s syndrome, or other systemic diseases that could interfere with reproductive function. In addition, patients who had used medications known to affect hormonal secretion, including myo-inositol or metformin, within the three months preceding enrollment were excluded from the study.

### Randomization and Interventions

Participants were randomly assigned to receive either letrozole (LZ) with lifestyle modification alone (LZ group) or letrozole combined with alpha-lipoic acid (ALA group) for three treatment cycles. Eligible patients were randomized in a 1:1 ratio according to a randomization sequence generated by the trial statistician using the Statistical Analysis System (SAS) version 9.4. Block randomization was applied to generate the allocation sequence. To minimize selection bias, random block sizes were used, and investigators were blinded to the size of each block.

In both groups, LZ 2.5 mg (Femara®, Novartis, Egypt) was administered orally once daily for five consecutive days, starting from day 3 to day 7 of either natural or progesterone-induced menstruation. In patients who failed to ovulate, the LZ dose was increased in subsequent cycles to 5 mg and then to 7.5 mg if necessary.

In the ALA group, participants additionally received ALA 600 mg three times daily (Thiotacid 600 Original®, EVA Pharm, Egypt). ALA administration started simultaneously with LZ and continued until the day of human chorionic gonadotropin (HCG) injection (Jannatifar et al. 2022). Patients were instructed to take ALA tablets 30 min before meals. Patient adherence to the prescribed medications was monitored by the clinical pharmacist through pill counting during follow-up visits as well as through telephone follow-ups. In cases where no dominant follicle was detected, ALA administration was discontinued for that cycle and resumed in the subsequent cycle following the same protocol.

Ovarian response was closely monitored, with ultrasound examinations performed every two days after the last dose of LZ. Ultrasound was used to assess the number and diameter of developing follicles as well as endometrial thickness. All ultrasound examinations were conducted by an experienced physician using standardized measurement protocols. Follicular diameter and endometrial thickness were recorded as quantitative parameters, and formal reports were generated for each assessment to ensure measurement consistency and minimize subjective interpretation.

At the same time, hormonal and metabolic parameters were measured. Hormonal assessments included serum estradiol (E2; pg/ml) and mid-luteal progesterone (mid-P; ng/ml). Metabolic parameters included fasting blood glucose (FBG; mg/dl), fasting insulin (FI; μIU/ml), and homeostasis model assessment of insulin resistance (HOMA-IR).

If no follicle larger than 10 mm was detected, along with an E2 level < 70 pg/ml and mid-P < 5.0 ng/ml two weeks after discontinuation of LZ, dydrogesterone (Duphaston®, Egypt) was administered to induce menstruation. Ovulation was confirmed either by ultrasonographic evidence of regression of a mature follicle measuring ≥ 18 mm or by a serum mid-P level > 5 ng/ml. All laboratory investigators responsible for biochemical analyses were blinded to the treatment allocation.

When at least one mature follicle measuring ≥ 18 mm was observed, 10,000 IU of HCG was administered. Couples were advised to have timed intercourse 36 h after HCG injection. A follow-up ultrasound examination was performed four weeks later to confirm clinical pregnancy. Patient adherence to treatment was also evaluated by counting the remaining LZ tablets during follow-up visits.

### Outcome variables

The primary outcome was the ovulation rate per woman, defined as the proportion of participants who achieved at least one ovulatory cycle during the study period. In addition, the cumulative ovulation rate (ovulation per cycle) was evaluated and defined as the number of cycles in which ovulation occurred relative to the total number of observed treatment cycles. The mono-ovulation rate was also assessed and defined as the proportion of cycles with a single mature ovum relative to the total number of ovulatory cycles.

Secondary outcomes included several reproductive, hormonal, metabolic, and ultrasonographic parameters. These comprised:

(i) **Clinical pregnancy rate**, defined as the ultrasound visualization of one or more gestational sacs with a detectable fetal heartbeat;
(ii) **Hormonal parameters**, including serum estradiol (E2) and mid-luteal progesterone (mid-P);
(iii) **Metabolic parameters**, including fasting blood glucose (FBG), fasting insulin (FI), homeostasis model assessment of insulin resistance (HOMA-IR), and body mass index (BMI). HOMA-IR, an index used to evaluate insulin resistance, was calculated using the formula:

$HOMA\;-\;IR\;=\;(fasting\;insulin\;mU/L\;\times\;fasting\;glucose\;mmol/L)\;/\;22.5$ (Madeira et al. 2008).

A HOMA-IR value of 2.71 was used as the cutoff for insulin resistance, as previously reported (Genazzani et al. 2012; Madeira et al. 2008).

(iv) **Ultrasound parameters**, including endometrial thickness.

### Statistical analysis

An intention-to-treat (ITT) analysis approach was adopted, whereby all randomized participants were included in the final analysis regardless of loss to follow-up. Statistical analyses were performed using SPSS software version 28 (IBM Corp., Armonk, NY, USA).

Quantitative variables were expressed as mean ± standard deviation (SD) and analyzed using a mixed-design analysis of variance (ANOVA) to evaluate the effects of time, treatment group, and their interaction. When a significant interaction effect was detected, simple main effects were further examined.

Comparisons between groups for continuous variables were performed using the independent samples t-test, with Bonferroni correction applied where appropriate to account for multiple comparisons. Categorical variables were presented as frequencies and percentages, and comparisons between groups were conducted using the Chi-square test or Fisher’s exact test, as appropriate. A two-tailed *P*-value < 0.05 was considered statistically significant.

### Sample size

The required sample size was estimated using the Power and Sample Size Program (PS), version 3.1.2. Based on a previously published study, the ovulation rate among patients with PCOS receiving L-carnitine combined with letrozole was reported to be 85%, compared with 65% among patients receiving placebo (Gharib 2019).

Using these estimates, a minimum sample size of 72 participants per group (total 144 participants) was required to achieve a statistical power of 80% with an alpha level of 0.05. To account for potential loss to follow-up, the calculated sample size was increased by 10%, resulting in a target enrollment of 80 participants per group (total 160 participants).

## Results

### Patient flow

**Fig. 1:**
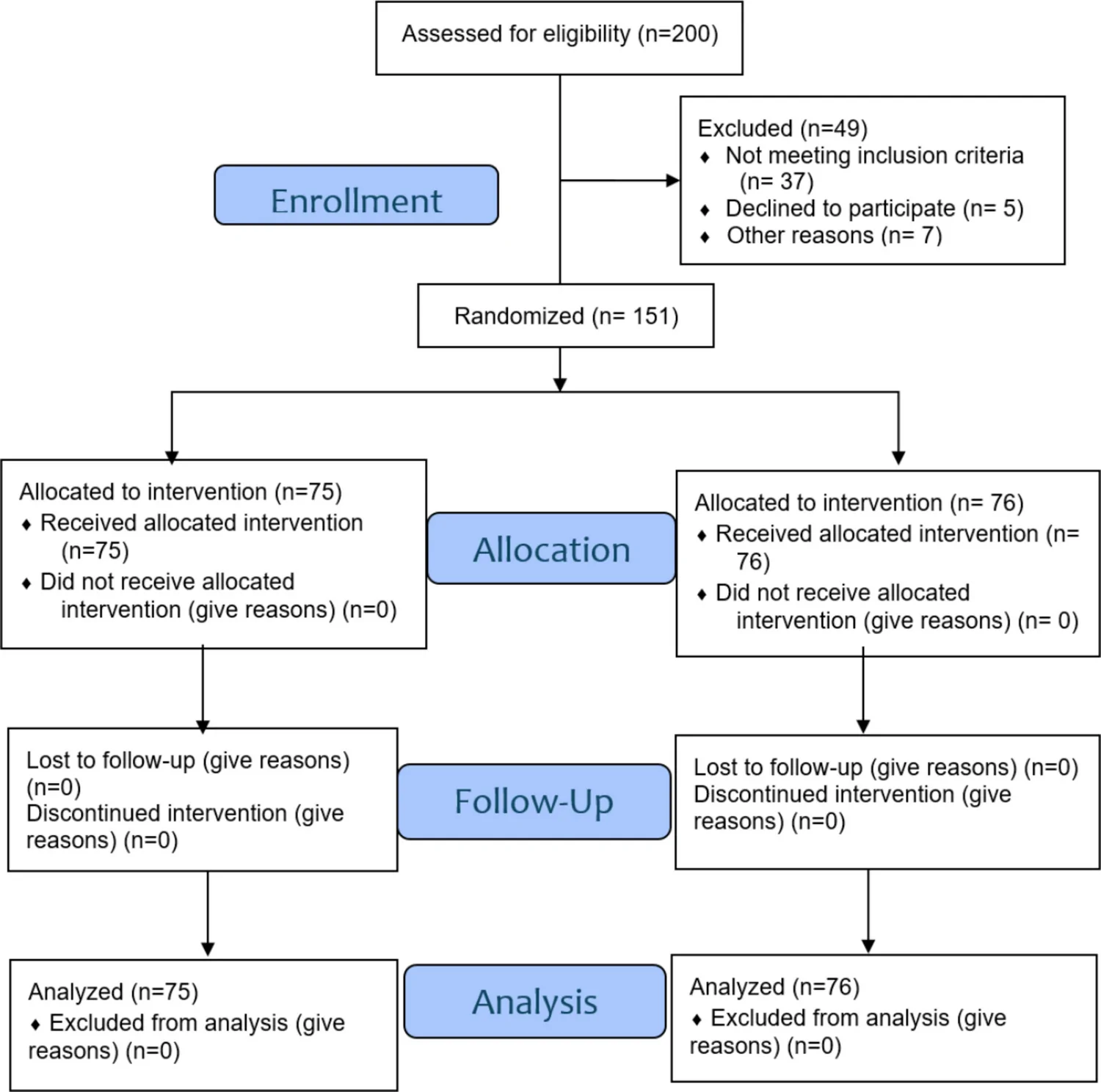
CONSORT flow diagram of participant recruitment and allocation

A total of 200 women with polycystic ovary syndrome (PCOS) were assessed for eligibility. Forty-nine patients were excluded due to not meeting inclusion criteria (*n* = 37), declining participation (*n* = 5), or other reasons (*n* = 7). The remaining 151 patients were randomized into two groups: the letrozole (LZ) group (*n* = 76) and the alpha-lipoic acid plus letrozole (ALA) group (*n* = 75). All randomized participants were included in the final intention-to-treat analysis. LZ: letrozole; ALA: alpha-lipoic acid (Fig. 1).

### Results of the analysis of the whole study groups data

#### Baseline characteristics

The baseline demographic and clinical characteristics of the study participants are presented in Table 1. At baseline, a statistically significant difference was observed between the two groups in fasting blood glucose levels (84.61 ± 8.88 vs 81.46 ± 10.26 mg/dL). However, other metabolic parameters, including HOMA-IR, BMI, and fasting insulin (FI), were comparable between the two groups. In addition, no significant difference was observed in the age of participants between the LZ group (26.39 ± 4.89 years) and the ALA group (25.79 ± 4.47 years) (*P* = 0.427).

**Table 1:** Baseline and post-treatment metabolic parameters of the study groups

Outcome	Group	Baseline Mean ± SD	Post-treatment Mean ± SD	Simple main effect of time (within group) *p*-value	Simple main effect of group at baseline *p*-value	Simple main effect of group at post-treatment *p*-value	Group × Time interaction *p*-value
**Fasting glucose (mg/dL)**	LZ	81.46 ± 10.26	80.44 ± 9.79	**0.002**	**0.046**	0.176	** < 0.001**
	ALA	84.61 ± 8.88	78.45 ± 8.15	** < 0.001**			
**Fasting insulin (mU/L)**	LZ	15.06 ± 5.68	14.31 ± 5.59	** < 0.001**	0.175	** < 0.001**	** < 0.001**
	ALA	13.83 ± 5.37	11.38 ± 4.42	** < 0.001**			
**HOMA-IR**	LZ	3.12 ± 1.44	2.91 ± 1.39	** < 0.001**	0.488	**0.001**	** < 0.001**
	ALA	2.96 ± 1.39	2.23 ± 1.09	** < 0.001**			
**BMI (kg/m**^**2**^**)**	LZ	25.84 ± 4.27	25.83 ± 4.20	0.604	0.805	0.498	** < 0.001**
	ALA	25.68 ± 3.70	25.39 ± 3.65	** < 0.001**			

Number of patients in LZ group, *n* = 76; Number of patients in ALA group, *n* = 75

Data are presented as mean ± SD

A two-way mixed-design ANOVA was performed with time (baseline vs post-treatment) as the within-subject factor and treatment group (ALA vs LZ) as the between-subject factor

Simple main effects of time represent within-group changes over time

Simple main effects of group represent between-group differences at each time point (baseline and post-treatment), based on Bonferroni-adjusted estimated marginal means

Statistically significant *p*-values are shown in bold

#### Primary outcomes (cumulative ovulation rate and ovulation rate).

The intention-to-treat (ITT) analysis demonstrated that the ALA group had a significantly higher cumulative ovulation rate compared with the LZ group (*P* = 0.035). In addition, the mono-ovulation rate was significantly higher in the ALA group than in the LZ group (*P* = 0.014). However, no statistically significant difference was observed in the ovulation rate per woman between the two groups (*P* = 0.194), as shown in Table 2.

**Table 2:** Ovulation, pregnancy, and hormonal data between ALA and LZ groups

Outcome	LZ group (*N* = 76)	ALA group (*N* = 75)	Unadjusted *p*-value	Adjusted *p*-value
**Ovulation rate**	61/76 (80.3%)	66/75 (88.0%)	0.194^a^	—
**Cumulative ovulation rate**	146/203 (71.9%)	153/166 (92.2%)	**0.035**^**a**^	—
**Mono-ovulation rate**	122/203 (60.1%)	120/166 (72.3%)	**0.014**	—
**Bi-ovulation rate**	21/203 (10.3%)	25/166 (15.1%)	0.173^a^	—
**Pregnancy rate**	26 (34.2%)	35 (46.7%)	0.119^a^	—
**Cumulative pregnancy rate**	26/203 (12.8%)	35/166 (21.1%)	**0.033**^**a**^	—
**Serum estradiol (pg/ml)**				
Cycle 1	190.4 ± 36.74	195.06 ± 18.53	0.409^b^	1.000
Cycle 2	188.06 ± 21.8	197.79 ± 21.69	**0.027**^**b**^	0.081
Cycle 3	189.44 ± 37.48	200.94 ± 16.47	0.067^b^	0.201
**Mid-luteal progesterone**				
Cycle 1	13.07 ± 3.33	13.84 ± 2.86	0.196^b^	0.588
Cycle 2	13.24 ± 3.07	14.28 ± 2.97	0.087^b^	0.261
Cycle 3	14.21 ± 3.56	15.69 ± 3.38	**0.049**^**b**^	0.147
**Endometrial thickness (mm)**				
Cycle 1	9.55 ± 1.48	9.82 ± 1.23	0.298^b^	0.894
Cycle 2	9.58 ± 1.11	10.04 ± 1.07	**0.036**^**b**^	0.108
Cycle 3	9.71 ± 1.70	10.37 ± 1.01	**0.030**^**b**^	0.090

Data are presented as mean ± SD or n (%). a: Chi-square test. b: Independent t-test

Bonferroni adjustment was applied to cycle-specific (ovulatory cycles) comparisons for estradiol, progesterone, and endometrial thickness across the three cycles. statistically significant *p*-values are shown in bold

#### Secondary outcomes

I. Pregnancy rate

The total pregnancy rate was higher in the ALA group than in the LZ group (46.7% vs 34.2%); however, this difference did not reach statistical significance. The highest pregnancy rates were observed during cycle 3 (24.5% vs 15.3%), followed by cycle 2 (18.5% vs 13.2%), and cycle 1 (13.3% vs 10.5%).

In contrast, the cumulative pregnancy rate per cycle was significantly higher in the ALA group compared with the LZ group (*P* = 0.033), as presented in Table 2.

II. Hormonal parameters

Hormonal parameters were analyzed only for ovulatory cycles. Within the LZ group, a significant difference across the three treatment cycles was observed only for mid-luteal progesterone (mid-P) levels (*P* = 0.016), with a significant increase detected in cycle 3.

**Fig. 2:**
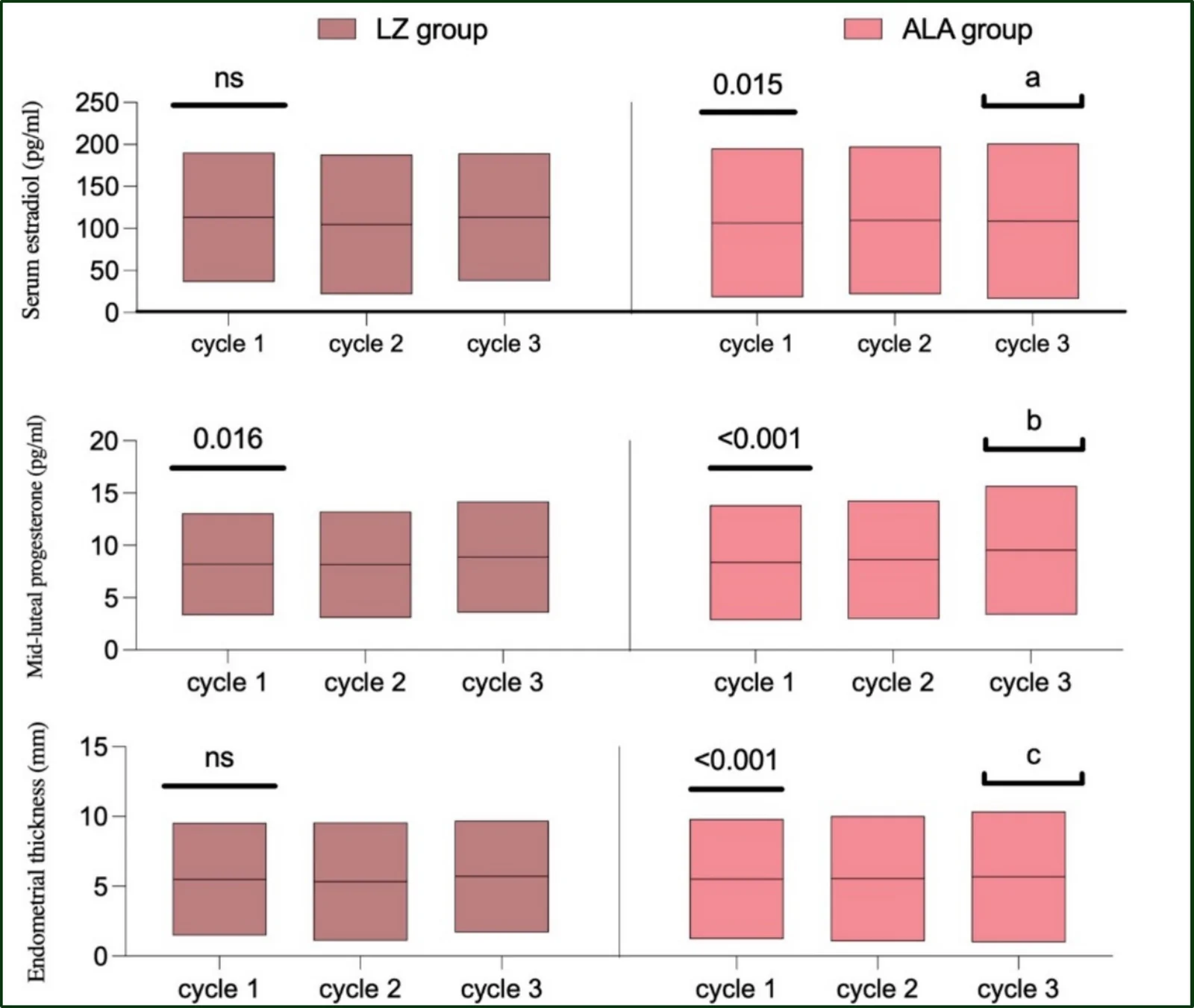
Changes in hormonal parameters and endometrial thickness across three ovulation induction cycles in the LZ and ALA groups. (**a**) Serum estradiol (E2) levels showed no statistically significant change across cycles in the LZ group, whereas the ALA group demonstrated a significant increase over time (*p* = 0.015), suggesting improved follicular development and ovarian steroidogenesis. (**b**) Mid-luteal progesterone levels increased significantly across cycles in both groups, with a more pronounced rise in the ALA group (*p* < 0.001), indicating improved luteal phase function and corpus luteum activity, which are critical for endometrial receptivity and early pregnancy maintenance. (**c**) Endometrial thickness did not significantly change in the LZ group but increased significantly in the ALA group (*p* < 0.001), suggesting improved endometrial development

In the ALA group, serum estradiol (E2), mid-P, and endometrial thickness showed significant variation across the three cycles (*P* = 0.015, *P* < 0.001, and *P* < 0.001, respectively), as illustrated in Fig. 2.

Between-group comparisons of ovulatory cycles revealed numerically higher values of serum E2, mid-P, and endometrial thickness in the ALA group compared with the LZ group. However, after applying Bonferroni adjustment for multiple comparisons, none of these differences remained statistically significant (Table 2).

III. Metabolic parameter

The mixed-design ANOVA demonstrated a statistically significant group × time interaction for all evaluated metabolic parameters, including fasting blood glucose (FBG), fasting insulin (FI), HOMA-IR, and BMI (all *P* < 0.001), indicating different temporal changes between the ALA and LZ groups (Table 1).

Further simple main-effects analysis showed that FBG levels decreased significantly over time in both groups, with a significantly greater reduction observed in the ALA group (*P* < 0.001) compared with the LZ group (*P* = 0.002). At baseline, FBG levels were significantly higher in the ALA group (*P* = 0.046); however, this difference was no longer observed after treatment (*P* = 0.176).

For fasting insulin, a significant reduction over time was observed only in the ALA group (*P* < 0.001), whereas the change in the LZ group was not statistically significant (*P* = 0.175). Post-treatment FI levels were significantly lower in the ALA group compared with the LZ group (*P* < 0.001).

Similarly, HOMA-IR values decreased significantly over time in both groups (both *P* < 0.001), with a greater reduction observed in the ALA group. No significant difference between groups was detected at baseline (*P* = 0.488); however, post-treatment HOMA-IR values were significantly lower in the ALA group (*P* < 0.001).

Regarding BMI, a significant reduction over time was observed only in the ALA group (*P* < 0.001), whereas no significant change occurred in the LZ group (*P* = 0.604). Although no significant between-group differences in BMI were observed at either baseline or post-treatment, the significant group × time interaction indicates a greater longitudinal improvement in BMI with ALA supplementation (Table 1).

#### Safety profile between

The incidence of adverse events during the trial was comparable between the LZ and ALA groups. Evaluation of the reported side effects showed no statistically significant differences between the two groups for any of the recorded adverse events, as presented in Table 3.

**Table 3:** Safety profile parameters over treatment

Side effect	LZ group (*N* = 76)	ALA group (*N* = 75)	*P*-value^b)^
Nausea	10 (13)	15(20)	0.258^a)^
Stomach upset	6(7.9)	9(12)	0.399^a)^
Vomiting	3(4)	3(4)	1.000
Diarrhea and bloating	2(2.6)	2(3)	1.000
Constipation	4(5)	4(5)	1.000
pruritus	2(2.6)	2(3)	1.000
Rash	4(5)	5(7)	0.745
Headache	13(17)	13(18)	0.970^a)^
Arthralgia	5(6.5)	4(5)	1.000^a)^
Hot flashes	16(21)	15(20)	0.873
Night sweats	7(9)	7(9)	0.979^a)^
Fatigue	8(10.5)	10(13)	0.595^a)^

Values are presented as number (%). ^a)^Chi-square test; ^b)^Fisher exact test

### Results of the analysis of data for patients with IR

#### Baseline characteristics

The initial metabolic profile showed that fasting insulin (FI), fasting blood glucose (FBG), body mass index (BMI), and HOMA-IR were comparable between the two groups. In addition, no statistically significant difference was observed in the age of the participants between the LZ group (26.21 ± 4.66 years) and the ALA group (25.78 ± 4.35 years) (*P* = 0.683) Table 4.

**Table 4:** Metabolic data of the studied IR-subgroup

Outcome	Group	Baseline Mean ± SD	Post-treatment Mean ± SD	Simple main effect of time (within group) p-value	Simple main effect of group at baseline p-value	Simple main effect of group at post-treatment p-value	Group × Time interaction p-value
**Fasting glucose (mg/dL)**	LZ	89.68 ± 7.22	88.05 ± 7.46	** < 0.001**	0.456	**0.017**	** < 0.001**
	ALA	91.01 ± 8.12	83.52 ± 8.61	** < 0.001**			
**Fasting insulin (mU/L)**	LZ	19.86 ± 3.89	18.77 ± 4.28	** < 0.001**	0.124	** < 0.001**	** < 0.001**
	ALA	18.71 ± 2.29	15.26 ± 2.22	** < 0.001**			
**HOMA-IR**	LZ	4.38 ± 0.89	4.06 ± 1.01	** < 0.001**	0.352	** < 0.001**	** < 0.001**
	ALA	4.20 ± 0.77	3.15 ± 0.73	** < 0.001**			
**BMI (kg/m**^**2**^**)**	LZ	28.55 ± 3.56	28.51 ± 3.49	0.250	0.721	0.418	** < 0.001**
	ALA	28.30 ± 2.53	27.93 ± 2.55	** < 0.001**			

Number of patients in LZ group, *n* = 38; Number of patients in ALA group, *n* = 37

Data are presented as mean ± SD

A two-way mixed-design ANOVA was performed with time (baseline vs post-treatment) as the within-subject factor and treatment group (ALA vs LZ) as the between-subject factor

Simple main effects of time represent within-group changes over time

Simple main effects of group represent between-group differences at each time point (baseline and post-treatment), based on Bonferroni-adjusted estimated marginal means

Statistically significant p-values are shown in bold

#### Primary outcome (cumulative ovulation rate and ovulation rate)

Among patients with IR, the ALA group demonstrated a higher ovulation rate compared with the LZ group; however, this difference did not reach statistical significance (83.8% vs 68.4%, *P* = 0.119).

In contrast, the cumulative ovulation rate was significantly higher in the ALA group than in the LZ group (71% vs 57.1%, *P* = 0.032), as shown in Table 5.

**Table 5:** Ovulation, pregnancy, and hormonal data of the IR group

Outcome	LZ group (N = 38)	AL group (N = 37)	Unadjusted p-value	Adjusted p-value*
Ovulation rate	26/38 (68.4%)	31/37 (83.8%)	0.119ᵃ	—
Ovulation per cycle (cumulative ovulation rate)	64/112 (57.1%)	76/107 (71.0%)	**0.032ᵃ**	—
Total pregnancy rate	5 (13.2%)	10 (27.0%)	0.133ᵃ	—
Pregnancies per cycle	5/112 (4.5%)	10/107 (9.3%)	0.153ᵃ	—
Serum estradiol (pg/ml) –				
Cycle 1	189.42 ± 24.77	187.52 ± 6.16	0.755ᵇ	1.000
Cycle 2	178.95 ± 19.15	187.63 ± 9.50	0.062ᵇ	0.186
Cycle 3	170.02 ± 13.76	194.78 ± 7.70	** < 0.001ᵇ**	** < 0.003**
Mid-luteal progesterone (pg/ml)				
Cycle 1	13.25 ± 2.33	12.42 ± 1.26	0.160ᵇ	0.480
Cycle 2	12.07 ± 2.65	13.12 ± 1.34	0.099ᵇ	0.297
Cycle 3	11.86 ± 1.82	14.33 ± 1.02	** < 0.001ᵇ**	** < 0.003**
Endometrial thickness (mm) –				
Cycle 1	9.72 ± 1.29	9.19 ± 1.11	0.172ᵇ	0.516
Cycle 2	9.24 ± 0.92	9.65 ± 0.97	0.138ᵇ	0.414
Cycle 3	8.78 ± 1.06	10.02 ± 0.91	** < 0.001ᵇ**	** < 0.003**

Data are presented as mean ± SD or n (%). ᵃ^)^Chi-square test; ᵇ^)^Independent-samples *t*-test

Bonferroni adjustment was applied to cycle-specific comparisons of serum estradiol, mid-luteal progesterone, and endometrial thickness across the three cycles. Statistically significant *p*-values are shown in bold

#### Secondary outcomes

I. Pregnancy rate

Among patients with IR, no statistically significant differences were observed between the ALA and LZ groups in either the total pregnancy rate or the cumulative pregnancy rate, with *P* = 0.113 and *P* = 0.153, respectively (Table 5).

II. Hormonal parameters

In cycle 3, serum estradiol (E2), mid-luteal progesterone (mid-P), and endometrial thickness were significantly higher in the ALA group compared with the LZ group (IR subgroup). The values were 194.78 ± 7.7 vs 170.02 ± 13.76 pg/ml (*P* < 0.003) for E2, 14.33 ± 1.02 vs 11.86 ± 1.82 pg/ml (*P* < 0.003) for mid-P, and 10.02 ± 0.91 vs 8.78 ± 1.06 mm (*P* < 0.003) for endometrial thickness after applying Bonferroni adjustment (Table 5).

Within the LZ group (IR subgroup), a significant decrease in serum E2, mid-P, and endometrial thickness was observed across the three treatment cycles (*P* = 0.005, *P* = 0.043, and *P* = 0.002, respectively).

**Fig. 3:**
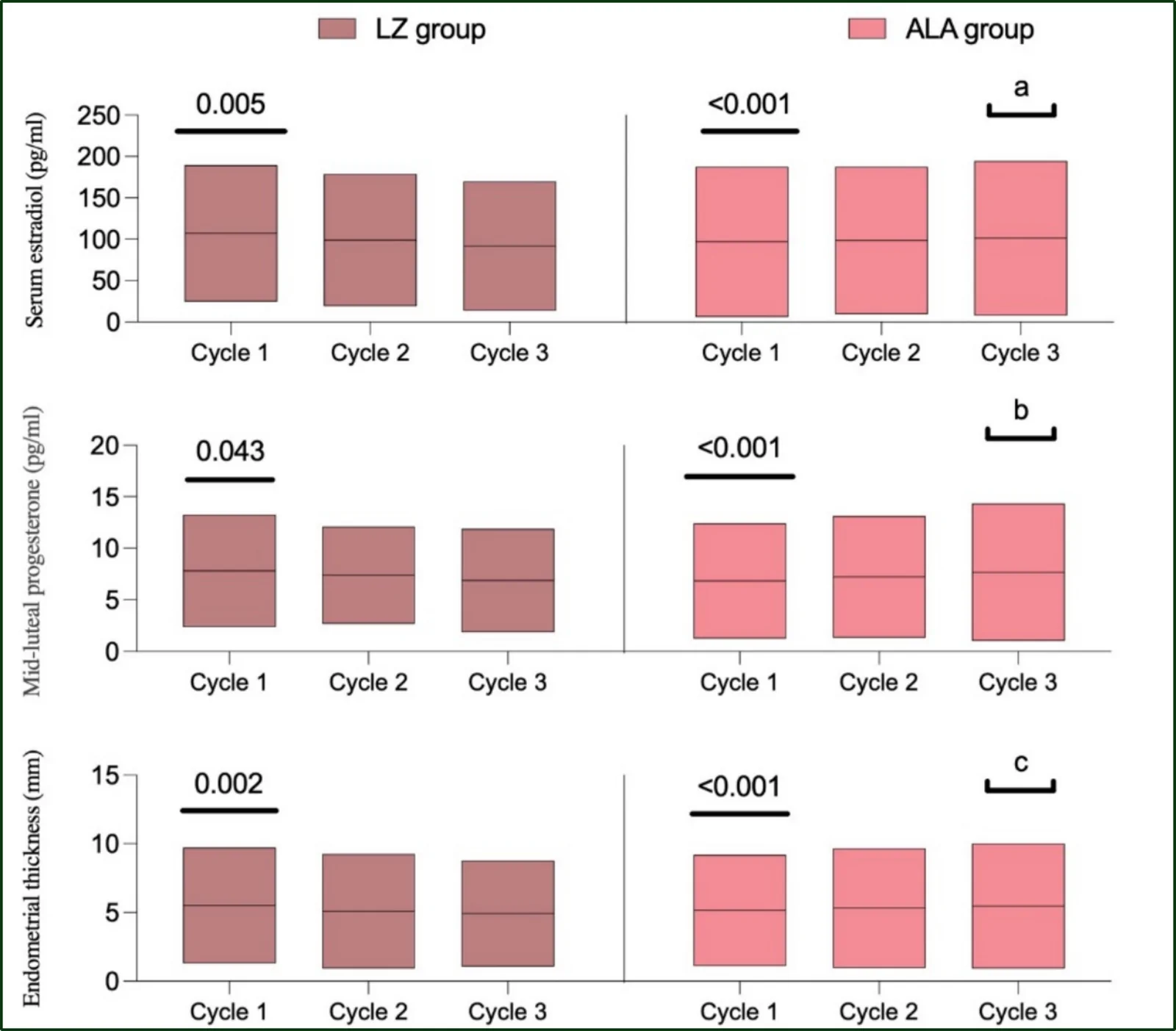
Hormonal and endometrial changes across ovulation induction cycles in PCOS patients with insulin resistance. (**a**) Serum estradiol levels increased significantly across cycles in both treatment groups, with a greater increase observed in the ALA group, reflecting improved follicular growth and ovarian steroidogenesis. (**b**) Mid-luteal progesterone levels showed progressive elevation in the ALA group compared with the LZ group, suggesting enhanced luteal phase function. (**c**) Endometrial thickness increased significantly across cycles in the ALA group, indicating a more favorable endometrial environment for embryo implantation

In contrast, within the ALA group (IR subgroup), serum E2, mid-P, and endometrial thickness differed significantly across the three cycles (*P* < 0.001). Specifically, these parameters increased significantly in cycle 2 compared with cycle 1 and further increased in cycle 3 compared with both cycles 1 and 2, as illustrated in Fig. 3.

III. Metabolic parameters

The mixed-design ANOVA revealed statistically significant group × time interactions for all evaluated metabolic parameters, including FBG, FI, HOMA-IR, and BMI (all *P* < 0.001), indicating different changes over time between the ALA and LZ groups among patients with IR (Table 4).

Further simple main-effects analysis demonstrated that all metabolic parameters improved significantly over time within each group (all within-group P < 0.001), with the exception of BMI in the LZ group, which did not show a statistically significant change (*P* = 0.250).

At baseline, no statistically significant differences were observed between the two groups for any of the evaluated metabolic parameters. However, post-treatment comparisons revealed significantly lower FBG, FI, HOMA-IR, and BMI values in the ALA group compared with the LZ group, as shown in Table 4.

## Discussion

Despite numerous studies investigating anovulation associated with polycystic ovary syndrome (PCOS), it remains a challenging condition in infertility management (Urman et al. 2004). PCOS is associated with several physiological disturbances, including hormonal imbalances, increased oxidative stress, metabolic dysfunction, and reduced ovulation rates (Mumusoglu et al. 2020). These abnormalities often result in the production of a greater number of low-quality oocytes during ovulation induction, which may subsequently reduce fertilization and implantation rates, increase embryonic fragmentation, and elevate the risk of miscarriage (Wu et al. 2017). Several pharmacological agents, including clomiphene citrate (CC), metformin, ethinylestradiol, and cyproterone, have been used to improve reproductive outcomes in women with PCOS. However, these medications are frequently associated with adverse effects such as ovarian hyperstimulation syndrome (OHSS), gastrointestinal disturbances, alterations in glucose metabolism and lipid profiles, psychological symptoms, hair density reduction, visual disturbances, and allergic dermatitis (Legro et al. 2013; Lord et al. 2003; Sharpe et al. 1996). In recent years, letrozole (LZ) has been increasingly used for ovulation induction in women with PCOS, as it helps restore hormonal balance while reducing the risk of OHSS. Nevertheless, the efficacy of currently available pharmacological treatments may remain limited when used as monotherapy, particularly in addressing insulin resistance (IR) and oxidative stress, thereby supporting the need for combination therapeutic strategies.

Alpha-lipoic acid (ALA) is a potent antioxidant with anti-inflammatory and insulin-sensitizing properties. Owing to its antioxidant activity and regulatory role in steroidogenesis, ALA has been proposed as a potential adjunctive therapy for PCOS management (Elmosalamy et al. 2024). Several clinical studies have evaluated the effects of ALA in patients with PCOS and reported improvements in metabolic parameters and oxidative stress markers. However, these studies have demonstrated limited effects on hormonal parameters, which prompted the investigation of ALA in combination with ovulation-inducing agents to enhance therapeutic outcomes. Therefore, the present study aimed to evaluate the effect of combining ALA with LZ on ovulation induction in women with PCOS. To the best of our knowledge, this study is the first to investigate the addition of ALA to LZ therapy in infertile women with PCOS and to evaluate its effects on ovulation rate, hormonal parameters, pregnancy rate, and metabolic profile.

The primary finding of this study demonstrated a significant improvement in the ovulation rate per cycle (cumulative ovulation rate) in the ALA group compared with the LZ group. From a clinical perspective, this finding is particularly important, as consistent ovulation is a key determinant of spontaneous conception in women with anovulatory PCOS. Although the ovulation rate per woman did not differ significantly between the groups, the cumulative ovulation rate across the three treatment cycles clearly indicates a sustained and progressive effect of ALA. These findings suggest that ALA may enhance the likelihood of conception by promoting a more consistent ovulatory response over time. Nevertheless, additional studies with longer treatment durations are required to further clarify the long-term impact of ALA on ovulation outcomes.

Furthermore, the mono-ovulation rate was significantly higher in the ALA group than in the LZ group (72.3% vs. 60.1%, *P* = 0.014). This finding suggests that ALA may promote the development of a single dominant follicle, which is considered optimal during ovulation induction. Achieving mono-ovulation may also reduce the risk of multiple pregnancies and associated complications, including OHSS.

These findings are partially consistent with the results reported by Morsy et al. who investigated the use of ALA in women with clomiphene citrate (CC)-resistant PCOS. In that study, ALA treatment improved ovulation and pregnancy rates compared with the control group receiving CC alone; however, the differences between the two groups did not reach statistical significance. Notably, the study included only 40 CC-resistant PCOS patients (Morsy et al. 2025). In contrast, our study evaluated the addition of ALA to LZ therapy in 75 PCOS patients, which may explain the more pronounced beneficial effects observed.

In the present study, serum estradiol (E2), mid-luteal progesterone (mid-P), and endometrial thickness were evaluated only during ovulatory cycles. The findings demonstrated improvements in hormonal parameters between the study groups. Serum E2 levels increased significantly across the three treatment cycles in the ALA group (*P* = 0.015), whereas only a slight and non-significant increase was observed in the LZ group. Although E2 levels were numerically higher in the ALA group compared with the LZ group, the difference between the groups did not reach statistical significance.

Elevated oxidative stress in women with PCOS may create a hostile ovarian microenvironment, impairing follicular development and oocyte maturation. Previous studies have demonstrated that ALA therapy can improve the intra-ovarian redox balance and modulate the gene expression of steroidogenesis pathways. Additionally, ovarian histomorphometric analysis has shown significant improvements following ALA treatment, accompanied by reduced immunological expression of caspase-3 in granulosa cells (Elmosalamy et al. 2024). By reducing oxidative stress, ALA may help create a more favorable ovarian environment, thereby enhancing ovulation efficiency. The observed increase in E2 levels in the present study, particularly in the ALA group, may therefore reflect improved follicular development, enhanced oocyte quality, and a healthier ovulatory response. Supporting this observation, a systematic review reported that higher levels of serum and follicular antioxidants are associated with improved oocyte maturation and quality (Murri et al. 2013).

Despite these promising findings, they should be interpreted with caution, as hormonal analyses were limited to ovulatory cycles only. Therefore, larger studies investigating this therapeutic combination are warranted to confirm these results.

Beyond ovulation outcomes, the findings of the present study demonstrated that the ALA group achieved a significantly higher cumulative pregnancy rate per cycle. This improvement was accompanied by a notable increase in mid-luteal progesterone (mid-P) levels across the three treatment cycles in the ALA group. Adequate progesterone secretion from the corpus luteum during the luteal phase plays a crucial role in establishing endometrial receptivity, which is essential for successful embryo implantation and maintenance of early pregnancy. In addition, increased levels of reactive oxygen species (ROS) or reduced antioxidant capacity have been associated with fertilization failure, implantation failure, and miscarriage (Sugino et al. 2004). Therefore, the observed improvement in reproductive outcomes among patients receiving ALA may be attributed to enhanced luteal function, reflected by higher mid-P levels, as well as the antioxidant properties of ALA. These findings suggest that ALA supplementation may improve reproductive outcomes in women with PCOS. Nevertheless, extending the duration of ALA administration in future studies may further enhance pregnancy outcomes beyond the cumulative pregnancy rate observed in the present trial.

Regarding endometrial thickness, our analysis across the three treatment cycles revealed no significant change within the LZ group (*P* = 0.196). In contrast, a significant increase in endometrial thickness was observed in the ALA group across the three cycles (*P* < 0.001). Several antioxidant systems, including thioredoxin and superoxide dismutase, have been identified within endometrial tissues and are believed to play an important role in endometrial receptivity (Sugino et al. 2004). Experimental studies have also demonstrated that antioxidants can improve the endometrial environment by inhibiting programmed cell death and reducing DNA damage (Aten et al. 1994). The significant increase in endometrial thickness observed with ALA supplementation in the present study may therefore be explained by its ability to reduce oxidative stress, promote cellular proliferation, and decrease local inflammatory responses, ultimately creating a more favorable uterine environment for embryo implantation.

However, these findings should be interpreted with caution because the hormonal and endometrial analyses were restricted to ovulatory cycles only.

The findings of the present study are consistent with those reported in a clinical trial evaluating two doses of LZ for ovulation induction in women with PCOS. In that study, 67 PCOS patients were randomly assigned to two groups: arm 1 (30 patients) received 5 mg/day LZ, while arm 2 (37 patients) received 7.5 mg/day LZ for five days starting from day 3 of menstruation. No significant difference in endometrial thickness was observed between the two treatment arms (7.87 ± 1.67 vs 7.16 ± 2.04 mm) (Ramezanzadeh et al. 2011). These findings are consistent with the results of the present study, in which no significant changes in endometrial thickness were observed across the three treatment cycles in the LZ group.

Several studies have also evaluated the addition of antioxidant and anti-inflammatory agents to LZ therapy, including L-carnitine (Gharib 2019), coenzyme Q10 (CoQ10) (Nasrin et al. 2022), and N-acetylcysteine (NAC) (Mostajeran et al. 2018). The findings of the present study are consistent with these reports, as the addition of ALA as an antioxidant resulted in improvements in both ovulation outcomes and cumulative pregnancy rates in infertile women with PCOS. For example, Nasrin et al. reported significant improvements in ovulation and pregnancy rates with the addition of CoQ10 to LZ therapy (Nasrin et al. 2022). Similarly, Mostajeran et al. demonstrated that adding NAC to LZ improved ovulation rates and increased follicular size, which may enhance the probability of pregnancy (Mostajeran et al. 2018). Moreover, both Gharib and Nasrin et al. reported improvements in endometrial thickness following the addition of L-carnitine and CoQ10, respectively, to LZ therapy (Gharib 2019; Nasrin et al. 2022). In agreement with these findings, the present study demonstrated a beneficial effect of ALA supplementation on endometrial thickness.

It is noteworthy that in the aforementioned antioxidant studies, the study medications were administered continuously for at least three months. In contrast, in the present study, ALA was administered for a maximum of 15 days per cycle. Despite this relatively short duration of administration, ALA demonstrated beneficial effects, which may be attributed to its potent antioxidant and metabolic regulatory properties. Therefore, future studies investigating continuous ALA administration for a longer duration (e.g., three months) may provide further insights into its potential to produce even greater improvements in reproductive outcomes.

Alpha-lipoic acid (ALA), often referred to as the “universal antioxidant,” is considered one of the most potent naturally occurring antioxidants (Cheng and He 2022). It is endogenously synthesized in small quantities within the mitochondria of human and animal cells, where it serves as an essential cofactor for several mitochondrial enzyme complexes involved in energy metabolism. ALA is a strong antioxidant and an amphipathic molecule with notable anti-inflammatory properties (Evans et al. 2002; Shay et al. 2009). Both ALA and its reduced metabolite, dihydro-lipoic acid (DHLA), act as powerful antioxidants capable of scavenging reactive oxygen species (ROS) and regenerating other endogenous antioxidants, particularly vitamins C and E, by restoring them from their oxidized forms (Abraham Gnanadass et al. 2021; Ma et al. 2010). In addition to their antioxidant activity, ALA and DHLA inhibit inflammatory signaling pathways mediated by nuclear factor-kB (NF-κB) by preventing its translocation into the nucleus, thereby reducing the production of pro-inflammatory cytokines and exerting immunomodulatory effects (Scott et al. 1994; Ying et al. 2011; Ma et al. 2010).

It has been reported that women with PCOS exhibit alterations in immune system function (Wang et al. 2023). Several studies have also demonstrated that chronic low-grade inflammation plays an important role in the development of long-term metabolic and cardiovascular complications associated with PCOS (Abraham Gnanadass et al. 2021; Mancini et al. 2021; Rudnicka et al. 2021). The beneficial effects of ALA in patients with PCOS may therefore be explained by multiple mechanisms. First, ALA acts as a potent antioxidant that reduces oxidative stress. Second, it inhibits inflammatory pathways regulated by NF-κB by preventing its nuclear translocation. Third, ALA improves reproductive function while correcting metabolic disturbances by enhancing glucose uptake and significantly influencing insulin metabolic pathways (Guarano et al. 2023).

In addition to its therapeutic potential, ALA is well known for its favorable safety profile. A comprehensive safety evaluation demonstrated that ALA supplementation was not associated with significant adverse events across a wide range of clinical conditions. Importantly, no harmful effects were observed in subgroups of participants categorized according to smoking status, cardiovascular disease, diabetes mellitus, pregnancy, neurological disorders, rheumatic diseases, severe renal disease, or pediatric and adolescent populations (Fogacci et al. 2020).

In the present study, analysis of metabolic outcomes demonstrated that fasting insulin (FI) and HOMA-IR levels were significantly reduced in the ALA group compared with the LZ group after treatment. Although fasting blood glucose (FBG) levels also decreased, the difference between the two groups after treatment did not reach statistical significance. This may be explained by the baseline difference observed between the groups (P = 0.045). Moreover, within-group analyses demonstrated significant reductions in FBG, FI, and HOMA-IR across the three treatment cycles, with greater improvements observed in the ALA group. These findings provide strong evidence supporting the ability of ALA to reduce insulin resistance (IR), which represents a central pathophysiological feature in many PCOS phenotypes (Purwar and Nagpure 2022). Insulin resistance not only exacerbates hyperandrogenism but also directly contributes to the anovulatory phenotype observed in PCOS (Purwar and Nagpure 2022; Zhu et al. 2024). By improving insulin sensitivity, ALA may indirectly restore hormonal balance and consequently improve ovulatory function. Furthermore, the metabolic improvements observed in this study are consistent with previous research demonstrating the beneficial effects of ALA in several insulin-resistant conditions, including type 2 diabetes mellitus and PCOS (Ansar et al. 2011; Evans and Goldfine 2000).

In the present study, patients were recruited according to PCOS phenotypic characteristics, specifically based on the presence or absence of insulin resistance (IR). Patients with type 2 diabetes mellitus were excluded because they require treatment with metformin. During data analysis, we observed that patients with PCOS who had IR demonstrated markedly different outcomes compared with those without IR in both treatment groups. Therefore, these patients were analyzed separately as an IR subgroup (IR-group).

The primary effect of ALA in the IR subgroup was the improvement of metabolic parameters. After treatment, the ALA group within the IR subgroup demonstrated significantly greater reductions in fasting blood glucose (FBG), fasting insulin (FI), and HOMA-IR compared with the LZ group. These findings suggest that ALA acts as a potent insulin-sensitizing agent. Insulin resistance is considered a key contributor to hyperandrogenism and anovulation in PCOS phenotypes. By enhancing cellular sensitivity to insulin, ALA may interrupt the cycle of hyperinsulinemia, thereby reducing androgen production from theca cells and contributing to the restoration of normal ovulatory function.

These findings are consistent with those reported by Masharani et al., who conducted a 16-week trial involving six lean, non-diabetic women with PCOS treated with controlled-release ALA (600 mg). The study reported a 13.3% improvement in insulin sensitivity following treatment (Masharani et al. 2010). Similarly, Genazzani et al. administered 400 mg of ALA for 12 weeks to 32 overweight women with PCOS and elevated BMI and observed significant improvements in insulin sensitivity (Genazzani et al. 2024). In another study by Genazzani et al., ALA administration for 12 weeks in obese women with PCOS resulted in significant reductions in insulin levels, BMI, and HOMA-IR following treatment (Genazzani et al. 2018).

However, the present study was not specifically powered to evaluate metabolic outcomes in the IR subgroup. Therefore, although the observed findings in this subgroup are promising, they should be interpreted with caution.

Regarding reproductive outcomes, the ovulation rate per cycle reached statistical significance in the IR subgroup (71% vs. 57.1%, P = 0.032). This finding suggests that although the immediate effect of ALA on ovulation within a single cycle may be variable among patients with IR, continued treatment across three cycles results in a significantly higher cumulative likelihood of ovulation.

In contrast, total pregnancy rate, pregnancy rate per cycle, and ovulation rate per woman did not show statistically significant differences between the two groups in this IR-subgroup. The absence of statistical significance may be attributed to the relatively small sample size of the IR-subgroup, which may have reduced the statistical power required to detect differences in these outcomes.

With respect to hormonal and endometrial parameters, the ALA group demonstrated significant improvements in all assessed parameters by cycle 3 within the IR-subgroup, whereas the LZ group showed a gradual decline in these parameters. The progressive improvement observed across treatment cycles suggests a cumulative therapeutic effect of ALA. This pattern indicates that the benefits of ALA may require time to fully manifest, possibly reflecting the gradual restoration of cellular metabolic activity and ovarian steroidogenic function. Future studies specifically powered to evaluate hormonal outcomes in patients with IR are needed to confirm these findings.

Overall, the improvements observed in hormonal and endometrial parameters, particularly during the later treatment cycles and across different PCOS phenotypes, suggest that the beneficial effects of ALA may be cumulative, leading to progressive improvement in the uterine environment with longer treatment duration.

### Limitations and recommendations

The gradual improvement observed across the treatment cycles suggests that longer treatment durations may produce greater therapeutic benefits. Therefore, future studies should investigate the optimal dosage regimens and treatment duration of ALA in order to maximize therapeutic efficacy while maintaining safety. In addition, comparative studies evaluating ALA against established treatments such as metformin and other commonly used pharmacological agents for PCOS are warranted.

Further research is also needed to evaluate the potential effects of ALA on pregnancy outcomes after conception, including miscarriage rates and obstetric complications. Such investigations would provide valuable information for the comprehensive clinical management of women with PCOS.

The safety of ALA supplementation was supported by the findings of the present study, as no significant adverse effects were observed during treatment. However, confirmation of the long-term safety profile of ALA through studies involving larger populations and extended follow-up periods is necessary before recommending its routine clinical use.

Several additional limitations should be acknowledged. First, the open-label design of the study may introduce potential bias and should be considered when interpreting subjective outcomes. Second, the relatively short evaluation period of three treatment cycles may not fully capture the long-term therapeutic effects of ALA supplementation. Third, the sample size was limited to women with PCOS meeting specific inclusion criteria, and participants were recruited from a single clinical setting, which may limit the generalizability of the findings to the broader PCOS population with varying phenotypes and disease severity. Furthermore, although the subgroup analysis of patients with insulin resistance (IR) yielded important findings, the relatively small sample size within this subgroup may have reduced the statistical power to detect smaller but clinically relevant differences. Therefore, these results should be interpreted with caution.

## Conclusion

In conclusion, the findings of this study provide evidence that ALA supplementation may enhance the effectiveness of LZ for ovulation induction in women with PCOS. Improvements were observed in ovulation rates, pregnancy outcomes, hormonal parameters, and metabolic profiles, suggesting that ALA may target multiple pathophysiological mechanisms involved in PCOS. Moreover, ALA supplementation appeared to be safe and well tolerated, supporting its potential as an adjunctive therapy to improve both reproductive and metabolic outcomes.

Nevertheless, larger multicenter clinical trials are required to further evaluate the therapeutic potential of this combination strategy, optimize treatment regimens, and assess long-term outcomes before its routine clinical application can be recommended.

## Data Availability

All data generated or analyzed during this study are included in this published article.
